# Occupational Health and Safety Statistics as an Indicator of Worker Physical Health in South African Industry

**DOI:** 10.3390/ijerph19031690

**Published:** 2022-02-01

**Authors:** Oscar Rikhotso, Thabiso John Morodi, Daniel Masilu Masekameni

**Affiliations:** 1 Department of Environmental Health, Tshwane University of Technology, Private Bag X680, Pretoria 0001, South Africa; moroditj@tut.ac.za; 2 Occupational Health Division, School of Public Health, University of Witwatersrand, Johannesburg 2193, South Africa; daniel.masekameni@wits.ac.za

**Keywords:** compensation, hazard and risk identification, health and safety legislation, health hazard, occupational disease, regulatory inspection, surveillance

## Abstract

Operations in general industry, including manufacturing, expose employees to a myriad of occupational health hazards. To prevent exposure, occupational health and safety regulations were enacted, with both employers and workers instituting various risk reduction measures. The analysis of available occupational disease and injury statistics (indicators of worker physical health) can be used to infer the effectiveness of risk reduction measures and regulations in preventing exposure. Thus, using the READ approach, analyses of occupational disease and injury statistics from South African industry, derived from annual reports of the Compensation Fund, were conducted. The publicly available database of occupational disease and injury statistics from the South African general industry is unstructured, and the data are inconsistently reported. This data scarcity, symptomatic of an absence of a functional occupational disease surveillance system, complicates judgement making regarding the effectiveness of implemented risk reduction measures, enacted occupational health and safety regulations and the status of worker physical health from exposure to workplace hazards. The statistics, where available, indicate that workers continue to be exposed to occupational health impacts within general industry, notwithstanding risk reduction measures and enacted regulations. In particular, worker physical health continues to be impacted by occupational injuries and noise-induced hearing loss. This is suggestive of shortcomings and inefficiencies in industry-implemented preventive measures and the regulatory state. A robust national occupational disease surveillance system is a regulatory tool that should detect and direct policy responses to identified occupational health hazards.

## 1. Introduction

Economic sectors such as the manufacturing and utilities sectors are cardinal in that their products are used for daily human sustenance as well as for supporting national economies [1,2]. Certain chemicals, for instance, are applied in medicinal formulations for disease prevention and control in humans, fertilisers to increase crop yields and pesticides that reduce plant disease in agriculture [3], allowing scope for their existence in spite of their inherent hazards [4]. According to the International Labour Organisation [5], both sectors are also a major source of employment globally. Besides the noted societal benefits, workers in these sectors are exposed to inherent occupational health hazards [5]. The expansion of these sectors requires the assessment and management of health risks from exposure [3].

Historically, the various industrial revolutions have brought about advancements in the machinery and manufacturing processes, which were and still are used for the mass production of goods in factories [6,7]. Advancements in mechanisation have resulted in unsafe work conditions [6,8], which create occupational health hazards, directly affecting workers [9,10]. Unsafe work conditions manifest themselves in observed occupational diseases (ODs) such as phossy jaw, a result of phosphorus fume exposure and clay dust poisoning in potteries, all having deadly outcomes on workers [6]. According to the United Nations Human Rights Commission 2018 special report, workers continue to face challenges in inadequate protection standards, slow progress in exposure prevention, fragmented occupational and environmental health strategies and gaps in exposure monitoring and enforcement [11]. Currently, occupational hazards are no longer limited to factories and mines but are even found in office buildings [12]. The regulatory response for exposure to occupational health hazards was through the enactment of occupational health and safety (OHS) laws, exposure limits and regulations prescribing how these hazards should be controlled [13,14,15]. From a South African perspective, the non-exhaustive historical list of OHS legislation enacted for exposure prevention is highlighted in Appendix A. The development of OHS legislation in South Africa had a historical foundation on the Factories, Machinery and Building Works Act 1941 [16]. The non-exhaustive, historical views shown in Appendix A attest that OHS regulation has been continually developed and refined over the years in South African general industry. Appendix A also shows the commencement date and purpose of each legislative instrument.

The OHS legislation in Appendix A incorporates the primary (hierarchy of control), secondary (screening and periodic medical screening) and tertiary prevention (medical treatment) methods for health and risk hazards [2,17,18,19]. The OHS legislation in Appendix A is enforced on self-regulation and regulator-enforced regulatory approach [20,21]. In this regard, the Inspectors of the Department of Employment and Labour are tasked with the enforcement of legislative pieces on the one hand [13,21]. While on the other hand, employers implement these OHS laws as a demonstration of legal compliance [13]. Although the Department of Employment and Labour’s annual reports indicates that the OHS inspectorate is conducting enforcement and inspections, the reports are scant on specificity relating to the violated legislation [22,23,24] (Appendix A). The reporting of ODs for compensation, initiated by the regulated industry, is directed by the Compensation Fund, a separate reporting structure of the Department of Employment and Labour [13,25,26].

In addition to the highlighted legislative instruments, South Africa is also a signatory of the International Labour Organization (ILO) Convention 155, “Occupational Safety and Health Convention”. However, the ILO conventions and recommendations are not binding unless ratified by a member country [27].

Despite the positive advancement in human development brought by industry and the enactment of OHS legislation, workers have paid a supreme price for contracting ODs and incurring physical injuries resulting from workplace exposures [28]. In particular, the occupational injury and disease burden for workers in low- and middle-income countries continue to rise [29]. The severity of such health impacts remains difficult to predict, however, as they occur gradually and cumulatively in the case of ODs [30].

The reported injuries and ODs from industry point employers and regulators to specific focus areas for prevention [31]. Accordingly, the most commonly reported OD is noise-induced hearing loss (NIHL), a nagging burden for compensation systems worldwide [32]. In spite of a strong knowledge base regarding OD occurrence and workplace regulation, it is apparent that current injury and OD incidence rates indicate a lapse in the application of this knowledge. However, complications arise from emerging issues such as concurrent exposure to chemicals, heat and cold stress, noise and vibration [3]. To judge the effectiveness of enacted legislative instruments, a review of national injury and OD statistics was necessary to shed light on the subject matter. The long-term prevention of injuries and ODs in occupational health programmes comes from hazard identification and risk assessments [33,34,35,36,37,38]. The aforementioned scholarly paper reviews publicly available injury and OD statistics to infer the state of the health of the workforce within South African general industry. This inference indirectly attests to the effectiveness of OHS regulations enacted to prevent ODs and injuries.

## 2. Materials and Methods

### 2.1. Conceptual Framework

The OHS regulations in Appendix A have not fully eliminated workplace hazards; however, data from other countries indicate that the implementation of similarly structured regulations has reduced some workplace injuries and ODs, largely attributable to regulator enforcement and inspection [39,40,41]. This thus indicates the need for a strong labour inspection regime to ensure a high level of compliance on the part of the regulated industry [20]. In this regard, Gray and Jones [42] posit that an increase in the number of regulatory inspections conducted in the United States’ manufacturing sector has resulted in a decrease in the number of citations and worker exposure levels.

**Figure 1:**
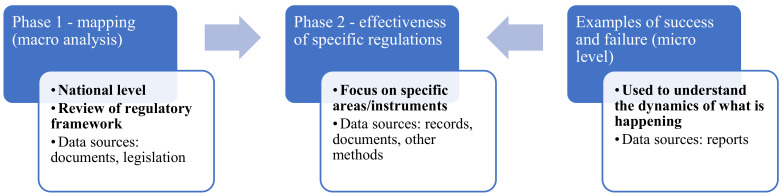
Conceptual framework adopted [43].

The microanalysis of OD and injury reports and the statistics released by the relevant institutions becomes necessary in order to gain insight into the subject matter. From a South African perspective, annual reports of the Compensation Fund, where available, are applicable. The conceptual framework employed for this review, adapted from Hongoro and Kumaranayake [43], is shown in Figure 1.

The observed low OD and injury incidence rates would infer the effectiveness of the OHS legislation in relation to the total employed population since inception. The absence of a functioning OD and injury surveillance can, however, make such adjudication cumbersome. This review focused on the microanalysis of annual reports of the Compensation Fund for injury and OD statistical information relevant for this study.

### 2.2. Search Strategy

#### 2.2.1. Occupational Disease and Injury Statistics

An online search for the annual reports of the Compensation Fund from 2002 to 2020 was conducted. Historically, these annual reports have provided high-level summaries of total OD and occupational injury counts with limited detail in regards to specifics, such as industry origin, race and gender; this is against a backdrop of an absent national OD and injury surveillance system.

#### 2.2.2. Employment Statistics

Against the backdrop of an absent national OD and injury surveillance system, an online search of the employment statistics from Statistics South Africa was used to shed light on the contributory factors of workforce characteristics and total employment in resultant OD and injury statistics. The employment statistics covered the period from the year 2001 to December 2019. The lag in the statistics relates to the retrospective nature of such data.

### 2.3. Document Analysis

**Figure 2:**
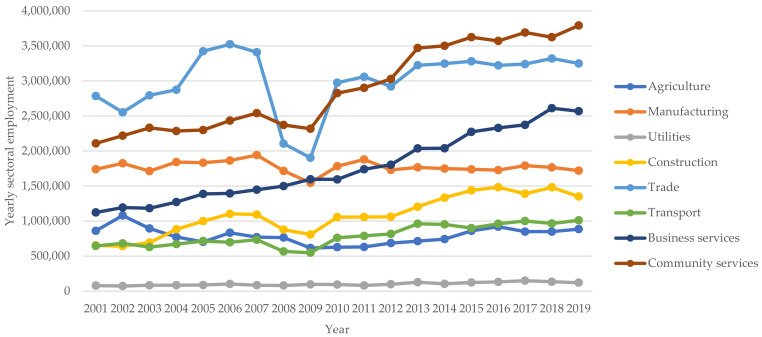
Sectoral employment in South Africa (excludes mining, private households).

**Figure 3:**
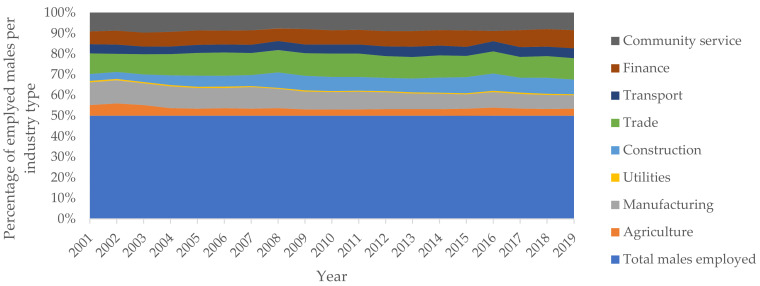
Male employment in South Africa (excludes mining, private households).

**Figure 4:**
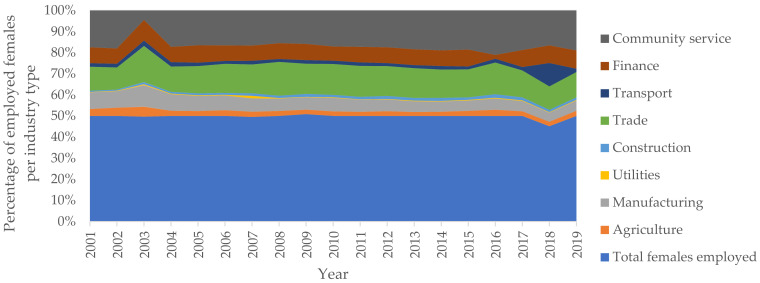
Female employment in South Africa (excludes mining, private households).

With a view of extracting meaning from the retrieved reports, document analysis was employed as a qualitative research method [44], with the perspective of obtaining quantitative data on OD and injury and employment statistics. Bowen [44] conceptualised document analysis as a research method that can be systematically applied in the evaluation or review of documents in print or electronic form. Document analysis enables the study to extract meaning from the data through examination and interpretation, which subsequently leads to a greater understanding of the subject matter [44]. Within the context of scientific research, document analysis denotes the analysis of any written material containing information about the topic of concern [45]. The READ approach [46] to document analysis was used in extracting meaningful data from the enrolled reports. The steps of the READ approach are: (1) readying the materials, (2) extracting the data, (3) analysing the data and (4) distilling the findings [46]. The READ approach to document analysis has been previously used in [47]. Figure 2, Figure 3 and Figure 4 and Appendix B present the results derived through the READ approach.

## 3. Results

The literature search returned results that limited the data in this study to annual reports published by the Compensation Fund. In general, the data was fragmented, difficult to locate and differed structurally, year to year. Reports relevant data to the study covered the years 2001 to 2019. No data for the reporting periods of 2014 and 2015 were located within the reports or any other public sources.

Occupational accidents, including both ODs and injuries, are reported to the Compensation Fund in line with the requirements of Section 24 of the Occupational Health and Safety Act [13], as well as Regulation 8 of the General Administrative Regulations [26]. Broadly, compensated injuries and ODs are regulated through the Compensation of Occupational Injuries and Diseases Act within South African general industry [25].

Due to an apparent absence of a national occupational disease and hazard surveillance system, statistical information from Statistics South Africa provides useful information on employment patterns. Thus, a background analysis of the number of workers employed in general industry was pertinent for this study.

### 3.1. Employment and Exposure Trends in South Africa

Statistics South Africa publishes quarterly labour market conditions for the country. In this regard, Figure 2 provides an overview of the sectoral employment in South Africa, whose occupational accidents are reported to the Compensation Fund for compensation purposes, covering the period between the fourth quarter of 2001 to the fourth quarter of 2019. Whereas Figure 2, Figure 3 and Figure 4 provide the gender and population group profiles of the workers employed in sectors indicated in Figure 2.

Figure 2 shows that between 2001 and 2019, trade and community services provided the majority of employment for the South African workforce. Employment from the manufacturing, utilities, construction, agriculture and transport sectors remained stable for the quoted period. The business sector (finance) did, however, see an increase in employment trends during the same period. The sectoral employment in Figure 2 provides evidence of employment shifts in the number of workers exposed to prevailing occupational health and safety hazards per industry type. Thus, the trade and community services industries constitute the highest number of workers exposed to inherent occupational health and safety hazards. The severity and scale of these hazards, however, differ from industry to industry, with the manufacturing sector cited as the most hazardous [10].

Figure 3 and Figure 4 show the gender distribution of the workers in each sector. The construction, manufacturing and trade industries employed more males combined. Meanwhile, the community service and finance industries provided more employment, combined, for females. Comparably, the South African labour market had more males in employment compared to females. This has historic underpinnings, wherein specific jobs were exclusively reserved for males. Anecdotally, males are the gender whose physical health from workplace exposure is most impacted by occupational hazards inherent in each respective industry under consideration.

On a different perspective of race at South African workplaces, the country’s labour laws differentiate the racial profile into Black African, White, Coloureds and Indians/Asians [48,49]. Generally, the South African population profile is predominantly Black African, with Whites, Coloureds and Indian/Asians making up the other percentage. This skewed demographic profile has also translated into workplace demographic disparities, wherein Black Africans constitute 70% of the employed population. Other race groups such as White, Coloured and Indians/Asians constitute 18%, 7% and 4% of the employed population, respectively. Anecdotally, Black Africans are the most exposed population group whose physical health from workplace exposure is greatly impacted by occupational hazards. However, exposure to inherent occupational health and safety hazards will largely depend on the industry type and tasks performed in the main. From a historic perspective, the white population exclusively occupied reserved managerial or supervisory positions, often seen as having less exposure compared to the physical tasks associated with manual work assigned to Black Africans, Coloureds and Indians/Asians [50,51].

### 3.2. Occupational Accidents in South Africa

The employment statistics for general industry and the total reported occupational accidents, including injuries and ODs, are shown in Appendix B. Between 2001 and 2019, a 19-year view, 3,852,071 occupational accidents were reported for compensation consideration, 3,808,177 of which were occupational injuries and 44,014 were ODs. The source industries of these occupational accidents are shown in Figure 2, and were reported to the Compensation Fund.

The total number of occupational accidents for the considered periods show that occupational injuries far outweighed ODs and closely tracked the total reported accidents. In general, ODs have both short and long latency, whereas occupational injuries are acute and have short-term latency [9]. The occupational injuries and OD disparity are unsurprising when considering the latency periods associated with each accident type. Notably, however, the total reported ODs excludes musculoskeletal disorders, an outcome of legal mechanisations in the administration of the Compensation for Occupational Injuries and Diseases Act [25]. Comparably, the lowest number of occupational accidents were reported at 81,995 in 2019, concurrent with a decrease in employment numbers within the manufacturing and construction sectors, as shown in Figure 2. The highest peak of reported accidents was recorded at 310,710 during 2013, concurrent with an increase in employment numbers from the agriculture, transport, construction and community service sectors. Of the periods under consideration, accident declines were noted during the reporting years of 2002, 2006, 2011, 2014, 2015 and 2019.

The data also shows that the majority of workers, in general, have jobs with low exposure and risk probability. In spite of the delayed latency of ODs, this is unsurprising as the dominant industries providing major employment for the South African labour market are trade, finance and community services. Business activities conducted in these sectors expose workers to mainly sedentary and ergonomic health and safety hazards. This, therefore, implies that the source industries for the reported occupational accidents include agriculture, manufacturing, construction and transport. However, in the absence of a national occupational disease and injury surveillance system capturing these indicators, such disambiguation is impossible.

The annual compensation fund reports considered in this study do not specify the nature of the specific occupational injuries reported, thus highlighting the paucity of such data on the one hand. Whilst, on the other hand, OD types were specified, though inconsistently, on some of the annual reports under consideration.

### 3.3. Occupational Diseases in South African General Industry

Table 1 differentiates specific ODs as a fraction of occupational accidents, as alluded to in Section 3.2. These ODs, accepted and subsequently compensated, cover diverse exposures resulting from workplaces that have physical, chemical and biological hazard types. These hazard types are regulated through the Environmental Regulations for Workplaces, Noise-Induced Hearing Loss-, Hazardous Chemical Agents- and the Hazardous Biological Agents-Regulations; legislative instruments reported in Appendix A [33,34,35,36,37,38]. The data in Table 1 show that workers continue to be exposed to physical, chemical and ergonomic health hazard impacts in spite of enacted workplace regulations; reported in Appendix A.

Of the compensated ODs, NIHL, a result of exposure to unprotected excessive noise levels, had the highest incidence in proportion to other diseases for the reporting periods, 2001 to 2019. All the recorded ODs show a decreasing trend for the covered period. Due to the lack of publicly available scientific literature investigating the effectiveness of the various legislative instruments in South Africa, it remains difficult to determine whether the OD decline can be attributable to OHS laws in Appendix A. Furthermore, it is inconceivable that legislation on its own has had such an impact on the decline in OD trends without effective enforcement and inspection regime [39,40,41], constantly cited as a hindrance to achieving the goals of OHS laws in most underdeveloped and developing countries.

No data were available in regards to OD specificity for the reporting years 2010 to 2013, as well as general data for all accidents for the periods 2014 to 2015. These ODs are preventable [52]. Comparably, the nature, cause and sources of occupational injuries remain unknown to the general public.

**Table 1:** Compensated ODs in South Africa [23,24,53,54,55,56,57,58].

Occupational Disease	Year																	
	2001	2002	2003	2004	2005	2006	2007	2008	2009	2010	2011	2012	2013	2014–2015	2016	2017	2018	2019
Noise-induced hearing loss (NIHL)	1465	1952	2549	2724	1823	3228	2644	785	1123	-	-	-	-	na	145	279	249	118
Tuberculosis of the lungs (in health care workers)	211	500	384	384	323	119	69	54	223	-	-	-	-	na	141	184	257	191
Occupational skin diseases	217	203	203	227	203	204	142	92	45	-	-	-	-	na	-	-	-	-
Pneumoconiosis	193	182	302	189	109	261	172	102	87	-	-	-	-	na	-	-	-	-
Occupational asthma	104	168	214	165	103	12	109	80	59	-	-	-	-	na	24	28	27	20
Mesothelioma	201	20	17	28	16	47	29	22	12	-	-	-	-	na	-	-	-	-
Irritant induced asthma	-	-	-	7	16	12	6	39	33	-	-	-	-	na	-	-	-	-
Lung cancers	-	-	-	4	1	8	9	5	3	-	-	-	-	na	-	-	-	-
Chronic obstructive airways disease	-	-	-	17	13	30	10	15	12	-	-	-	-	na	-	-	-	-
Diseases caused by chemical agents	-	-	-	69	15	35	323	105	98	-	-	-	-	na	-	-	-	-
Diseases caused by physical agents, excluding noise	-	-	-	-	-	14	10	27	31	-	-	-	-	na	-	-	-	-
Diseases caused by biological agents, excluding TB	-	-	-	75	228	275	144	75	63	-	-	-	-	na	-	-	-	-
Chemical exposure	-	-	-	-	-	-	-	-	-	-	-	-	-	na	64	68	35	41
Others ^1^	970	1664	1349	1469	972	105	21	12	45	-	-	-	-	na	-	-	-	-
Total	3361	4689	5018	5358	3822	4564	3720	1443	1895	1111	1475	2579	2579	na	374	559	568	370

^1^ OD name not specified. na Not available.

The source industries of the ODs in Table 1 are indicated in Figure 2, including activities as defined per the Statistics South Africa industry classification of economic activities [59].

## 4. Discussion

This study evaluated the status of worker physical health in South African general industry through the analysis of OD and injury statistics reported by the Compensation Fund. Additionally, employment statistics published by Statistics South Africa were considered in the analysis providing contextual background to ensuing discussions. The quoted statistics from South African general industry show that injuries far outweigh ODs, in part due to their short latency periods. The long latency of ODs, on the other hand, offers governments, employers and workers worldwide room for their elimination through the establishment of effective OD and hazard surveillance systems [60]. In this regard, the OD morbidity rates can direct policymakers to specific occupational hazards requiring urgent policy intervention.

The OD statistics for the quoted periods showed a declining trend. This decline and noticeable aggregate low levels of OD morbidity levels have seeming peculiarities, however, which relate to the increasing proportion of workers potentially exposed to occupational health hazards against registered ODs and injuries [61]. The statistics indicate that increases in employment have had a corresponding increase in occupational injuries, concurrent with a decline in OD morbidity. Thus, the noted increase in the number of occupationally exposed workers may well underestimate unreported cases [62,63]. This is symptomatic of a developmental state of occupational health services [63], South Africa included.

Another peculiarity in the noted declining OD morbidity stems from the noted backlog and carry-over accidents in some of the analysed Compensation Fund reports [53,54,55,64]. Once finalised, these carry-over accidents may reverse the current “seeming” declining outlook. The reduction in NIHL cases, in particular, is questionable amidst the dearth of information on studies relating to the prevalence of NIHL and industry noise reduction efforts in South African industry. The available field studies [65,66,67] indicate that workers are still exposed to excessive noise levels. Specifically, the implementation of hearing conservation programmes in South Africa is largely fragmented [67,68]. Further doubt is cast on the reported declining NIHL cases, from a legislative perspective, as the country has had no changes in the legislative framework governing noise exposure at work since 2003, for which the decline can be ascribed.

Undoubtedly, when OHS laws, such as those in Appendix A, are fully implemented by employers and enforced by inspectorates, a reduction in OD and injury rates can be expected [69]. However, Table 1 indicates that employer compliance and regulatory enforcement have not entirely eliminated OD and injury morbidity. From this perspective, evidence of enforcement and inspection exists in South Africa via the Department of Employment and Labour annual reports detailing all the department’s activities. However, these reports are adjudged as scant in detail relating to the specific violations noted during enforcement and inspection activities. As an example, in the 2015/2016 annual report, 20,476 notices were issued to the industry for health and safety violations during 23,678 workplace inspections [22]. Although the decreasing trend in OD is positive and can somewhat be anecdotally ascribed to employer compliance and regulatory enforcement [39,41,69], ascribing the decrease entirely to compliance and regulatory inspection is currently a challenge. A challenge admittedly acknowledged by the Department of Employment and Labour relating to the country’s enforcement regime [70]. From a scientific and technical perspective, to ascribe the reduction in OD morbidity requires measurement of statistics comparing changes in OD rates for both inspected and non-inspected workplaces [71]; data currently absent in South Africa.

The declining trend in OD morbidity is a positive development; however, the above discourse indicates that South Africa needs to focus more energy on data collection initiatives related to occupational hazards, OD and injury morbidity. The discourse also indicates that the physical health and life of workers is still afflicted by occupational hazards and manifested by ODs and injuries. The full and realistic extent of the affliction, however, remains unknown due to the fragmentation in data collection and the paucity of publicly available statistics. To compound the problem, South Africa currently has an absent national occupational health and safety policy and strategy, a loophole acknowledged by the Department of Employment and Labour [70].

### 4.1. Employment and Exposure Trends in South Africa

The employment landscape in South Africa shows that community services and finance are the largest employers. However, the manufacturing, utilities, agriculture and trade sectors are often cited as the most hazardous [10], compared to office-based work associated with community service and finance. Employees at these industries are often exposed to physical, chemical and biological occupational health hazard types [10,19,72,73,74,75], which are all linked to the ODs in Table 1. The paucity of the Compensation fund-derived OD and injury statistics complicates the process of attributing the specific sectors from which these accidents emanated. With regard to the manufacturing sector, however, occupational health hazards continue to feature in the modern production processes employed in the various industry types [76]. The transfer of manufacturing to developing countries from developed countries, some with untreated occupational health hazards, consequently sees the cross-border transit of these hazards inherent of such processesWatterson [77]. Coupled with poor OHS regulation and enforcement [78,79,80], workers employed at these facilities in developing countries are severely impacted compared to developed countries [76,77]. The somewhat low impact of occupational hazards in developed countries has been credited to public awareness leading to protest as well as media publicitySellers and Melling [76].

The prevalence of exposure to occupational health hazards is, however, not limited to South Africa; Watterson [77] posited that Romanian industries had far worse and unacceptable working conditions relating to occupational hazards compared to their Hungarian and Slovenian counterparts. Similarly, industries in Latin American countries were also reported to have exposure to unacceptable working conditions [81].

Indigenous ethnic groups across a range of different countries, including Blacks and Hispanics, are the most exposed to these occupational hazards owing to task distribution and task segregation [82,83]. In South Africa, race at the workplace is topical and legally regulated due to past injustices [48,49]. The historic apartheid economic planning concentrated the manufacturing base mainly in Gauteng, Kwazulu-Natal and the western Cape Provinces. Due to the high concentration of the population in these three provinces, it is thus unsurprising that a 2017 Statistics South Africa report indicated that these provinces accounted for 63% of deaths reported in the country, some of which are due to accidental injury from exposure to occupational health hazards. The black population accounted for the majority of the reported deaths due to the high population proportion compared to whites. Of the reported deaths in the same report, accidental poisoning by and exposure to noxious substances; exposure to electric current, radiation and extreme ambient air; and contact with heat and hot surfaces were attributable to occupational health and safety hazards [84]. Combined, the formal employment sector in South Africa, excluding agriculture, employed some 15 million workers. Of this total, Black Africans, Coloureds and Indians/Asians, excluding whites, were at 11 million. Of the specific occupations, plant and machine operators, craft and related trade and elementary occupations accounted for approximately six million workers in direct contact with occupational health hazards in their daily tasks [85]. This, therefore, highlights the racial disparity in the distribution of occupations with subsequent exposure to occupational health and safety hazards in South Africa, an important consideration that any future hazard and disease surveillance system should account for.

The above discourse highlights that occupational race/ethnicity and gender segregation have consequential outcomes in the areas of hierarchal authority and wages [86], applicable to South Africa. As an example, a study conducted in New Zealand found that the ethnic Maori workforce was more likely to be exposed to chemical, physical, organisational and stress factors compared to the non-Maori workforce [83]. Eng et al. [83] postulated that the exposure disparity is also a result of over-representation within the manufacturing sector of the ethnic workforce in low-skilled manual jobs. Immigrant workers of different ethnicities in particular, due to their perceived low skill levels, are exposed to hazardous work such as handling toxic products [87,88], leading to high incidence OD rates [82]. This racial and ethnic disparity also extends to the biased reporting of occupational injuries and ODs. To this effect, Sabbath, Boden [89] and Ning, Zhou [88] postulated that the underreporting of occupational injuries is higher among black and migrant workers, leading to underestimations of injuries along racial lines. Murray [90], however, cautions that further studies are required to grasp the extent of racial/ethnic disparities on occupational health. Notwithstanding the discourse above, risk perception by these exposures is an important consideration in preventing ODs along racial and ethnic lines [75].

#### Occupational Health Hazards, Exposure and Latency

The main picture presented by the ODs in Table 1 confirms worker exposure and health impacts inflicted in South African general industry [91]. The ODs are attributable to exposure from physical agents such as noise [8,65,92,93], an array of hazardous chemical agents [92,94,95,96,97,98,99,100,101] and hazardous biological agents such as mycobacterium tuberculosis [92,102,103,104,105]. Tint [19] and Ashford [30] postulated, for instance, that a single worker is exposed to a combination or a part of these hazards, depending on the type of process and job category. The cumulative effects of some of these hazards, even at low exposure levels, can result in chronic health impacts [10,106].

Worker exposure to occupational health hazards is also commonplace in other developing countries [63]. On this point, a survey carried out by local health inspectors in Estonia revealed that 16% of industrial workers were exposed to chemical, physical and biological agents [19]. Physical hazards and hazardous chemical substances were also identified in an occupational hygiene survey of 99 small Finnish workplaces [73] and Sri Lankan medium- and large-scale industrial sectors [72]. The extent of these hazards varies depending on the industry type, locality and precautionary measures taken by the worker and the employer [107,108,109]. The extent of chemical hazards, for example, occurs due to unreacted feedstocks and other process inputs, such as fillers, stabilisers, pigments, inhibitors and initiators, all added to a finished product [110]. To compound the problem, there are in excess of 100,000 hazardous chemicals in various workplaces, with new chemicals introduced yearly, all affecting workers [111]. From another perspective, in China, for example, Brown [108] reported that approximately 60% of businesses have “minimal industrial safety measures”, such as employee training and personal protective equipment. Similarly, in the Philippines, specific OHS issues reported to be grossly violated or disregarded by most employers included occupational health and environmental control, general ventilation and personal protective equipment [112]. This, therefore, points to shortcomings in actions taken to provide a safe and healthy workplace [113]. Some of the reasons for the exposure of workers are related to worker oversight in safe operating procedures and neglect of using preventive safety equipment [114]. Therefore, workers have a moral duty of protecting themselves against risks and hazards [109]. Gerkin and Doyon-Martin [115] assert that national governments are to blame, along with corporations, for repeatedly creating workplace conditions that lead to routine violations of OHS regulations associated with occupational health hazards.

Given the context, it is apparent that a safe and healthy workplace is a fundamental worker right to physical health, which is somewhat threatened by occupational hazard presence [108,112]. This state of affairs shows that employers ought to disclose the health risks posed by hazards during pre-employment to afford workers an opportunity to make informed decisions in respect to compensation and performing dangerous work [116]. In this regard, employers and workers should understand that no amount of economic gain can compensate a workers’ death or serious injury [117].

From a different perspective, the ODs in Table 1 are a result of cumulative exposure at sufficient exposure doses with different latency periods. In this regard, certain respiratory and skin diseases have long latency durations compared to exposure to allergic chemical hazards, which may trigger immediate responses amongst exposed susceptible workers, regardless of whether exposure concentrations complied with set regulatory limits or not. In view of the long latency of ODs and the associated complex diagnosis of disease aetiology, it is thus apparent that hazard surveillance would allow for targeted intervention on the primary causes of the respective diseases [60]. The effectiveness of industry-implemented hazard prevention measures with long latency periods, as prescribed in OHS legislation worldwide, currently has limitations in historic biological exposure indices and exposure levels, and their linkages to future ODs remains elusive [118]—for chemical hazard types, for example. The enacted OHS legislation [118], technological advancements, improved work practices and a decreasing trend in exposure over the decades should, however, be lauded for its stated intention to eliminate ODs, though quoted statistics indicate that they have not entirely attained this intended goal [118,119].

### 4.2. Occupational Disease Trends in South African General Industry

The reported ODs in Table 1 are a result of statutory health surveillance for workers occupationally exposed to corresponding hazards in South African general industry [33,34,35,37,38,60]. In total, 43,485 ODs were reported and compensated for between the quoted periods of 2001 and 2019 in South African general industry, which showed a declining trend. Noise-induced hearing loss was by far the highest compensated OD in relation to the total proportion of reported diseases in South Africa, a trend also reported in the Russian Federation [120] and Brazil [63], and to a lesser extent in China [121]. Occupational disease morbidity and mortality indicate an urgent need for effective workplace exposure prevention programmes [122]. An active and effective OD surveillance system can be quick to address emerging mortality and morbidity trends [123]. These disease surveillance systems record both fatal and non-fatal sentinel ODs, which are linked to occupations.

Of the identified ODs, NIHL is the most pervasive and prevalent inherent of some processes in the manufacturing sector [73,124]. The manufacturing sector consequently has the highest number of noise-exposed workers [125,126]. Workers in different industries, such as coal-fuelled power plants, textile mills, chemical manufacturing plants and steel plants, are also exposed to noise levels above the regulated exposure limits during routine activities [19,74,112,126,127,128,129,130]. Maximum noise levels measured in these sectors can reach 120 dB [128] and contain different spectral frequencies [131]. Publicly available noise records show a general reduction in industry noise levels since the late 1970s [124,132]. However, statistics from the US Bureau of Labour Statistics and South African Compensation Fund indicate that NIHL incidence rates still remain high within the manufacturing sector, regardless of the noted reduction [23,24,53,54,55,56,57,129,133,134], as well as process automation in some operations [129]. Contrarily, in the United Kingdom, the NIHL incidence rate has remained consistently low [135], whereas hearing loss is much more prevalent in the Asian manufacturing sector [136].

In view of the above discourse, it is thus apparent that to detect the impact of occupational hazards on workers, a hazard and disease surveillance system is a necessity and should form part of a country’s capacity-building efforts in OD prevention efforts [119,137]. In South Africa, a data gap remains with regards to the availability of exposure data, national information on employment, total number of occupationally-exposed workers and corresponding exposure levels [119].

The reviewed South African compensation reports also indicate that the fund continually disburses finances to workers suffering from occupation-induced disabilities. No statistics are available that indicate the actual number of workers under this continuing compensation system. However, the annual costs related to disablement, as indicated on the compensation fund annual reports (where available), ranged between ZAR 1.8 to ZAR 2.5 billion for the reporting period 2001 to 2019. This, therefore, indicates the additional burden placed on the financials of the compensation in South Africa.

#### Occupational Diseases and Mortality

Exposure to some of the attributable occupational health hazards in Table 1, specifically carcinogens and poisons, has led to mortality and maiming of workers [112,121,138], the extent of which remains largely unknown in part due to an absent population-based occupational health surveillance system. To shed light on this matter, a 2017 Statistics South Africa report on mortality and causes of death in South Africa indicated that tuberculosis, a communicable disease, was the main underlying cause of occupation-related deaths amongst males [84], which, in general, has historical links to the mining sector. The report further stated that a total of 34,325 deaths were attributable to accidental injury or exposure [84]. This then highlights the importance of OD prevalence statistics and mortality rates in providing alternative information sources on the subject matter. In this regard, a case study analysing the mortality rate associated with pneumoconiosis in South Africa indicated that workers in the manufacturing sector had an odds ratio of 4.77. In particular, engineers and machinery mechanics were occupations with increased pneumoconiosis deaths, with an odds ratio calculated at 6.85 [100].

To highlight the prevailing mortality and morbidity trends associated with workplace exposure on an international scale, studies from the United States [139], China [138] and Italy [140] linked historic worker chemical exposure to mortality [139,140]. Of the physical stressors, analysis of statistics from the United States Bureau of Labour Statistics showed that heat exposure accounted for some 32% of exposure-related fatalities between 2000 and 2010 [122]. The increase in mortality rate points to priority areas of disease prevention [141].

Mortality and morbidity rates are themselves influenced by factors such as duration of employment at a job [142,143] and regional and lifestyle factors [143]. Amidst the above discourse, there is, however, a pressing need for methodological alignment for studies investigating exposure/outcome links [144], which can be fulfilled by a national OD and injury surveillance system. Mortalities and morbidities linked to historic workplace exposure indicate that afflicted workers are paying the ultimate price from these chronic and oft-cumulative exposures.

## 5. Conclusions

Despite the enactment of occupational health and safety legislation, some occupational health and safety hazards remain unabated, as it is an accepted reality that it is impossible to eliminate all occupational health hazards. Thus, hazardous workplaces and occupations should be prioritised for hazard control, training and occupational health services [90]. Workplace studies reporting noncompliance to hazards, such as noise, thermal stresses, vibration, electromagnetic fields, ionising radiation, chemical substances and dust should spring labour inspectorates into intensive enforcement activities [19].

As all occupational diseases and injuries are man-made and preventable [145], their prevalence thereof points to shortcomings in industry-implemented preventive measures, inclusive of health and safety laws, exposure limits and protective measures. Workers employed in industry bear the economic and disease burden associated with exposure. Workplace morbidity and mortality statistics in South Africa indicate that workers exposed to identified occupational hazards continue to pay the ultimate price through fatalities, for example, in cases of diagnoses related to carcinogens, apart from poisonings. Of the occupational diseases, noise-induced hearing loss incidence remains high in general industry amidst technological advancement in the design of quieter equipment; this finding is most revealing. Even though the impact of noise on workers and its control is well researched, exposure continues. Thus, innovative interventions to curb this problem are required [146]. Even though the quoted OD statistics indicate a declining trend, a welcomed development, such a decline has unresolved peculiarities.

A national hazard and disease surveillance system is a necessary policy tool that can inform efforts in worker protection, such as the identification of high-risk industries inclusive of those with high noise levels. The disease prevalence rates drawn from the system can be used by regulators for targeted intervention programmes [147,148]. Another governmental strategy can be the incentivisation of organisations to implement hazard control initiatives in exchange for a reduction in compensation fund contributions. The positive effects of this incentive scheme were reported by Elsler, Treutlein [149]. Conclusively, occupational health and safety within South African general industry require urgent reformation and improvements on a regulatory level. This is in view of the paucity of OD and injury statistics from the compensation fund.

Recommendation for the establishment of a national occupational disease and injury surveillance system

In South Africa, the responsible agency for repositing ODs is unclear, but is supposedly and generally accepted as a function of the Compensation Fund, which records ODs emanating from general industry. However, publicly available OD statistics are inconsistently reported with minimal detail. On the other hand, developed countries have national disease surveillance systems in place for collecting and disseminating OD statistics to inform policy intervention. A lack of, or an inefficient, OD surveillance system has been cited by Ding et al. [138] as a major stumbling block in disease prevention.

Against this backdrop, a systemic national occupational health surveillance system underpinned by preventive policies and registry systems is recommended to enable targeted intervention [137,150,151]. The system will collect, tabulate and interpret OD data, which will be a useful input in setting national priorities for the attainment of safe and healthy workplaces [152,153]. Available OD statistics in countries like South African general industry do not have limits to their usefulness. The system can be used to trend OD prevalence, aggregate sectors, categories of exposed workers and health outcomes [150,151,154]. Data derived from the surveillance system can further be used for different purposes in both the private and public sectors. As an example, the ensuing data can be used for formulating arguments for and against legislation impact, identification of areas requiring special emphasis by employers, development of regulatory policy, standards and guidance by government agencies, use by analysts and researchers in the evaluation of regulatory effectiveness and identification of factors associated with ODs amongst others [155]. An added benefit of the system is that it would also contribute to the prevention and management of the economic and societal burdens associated with ODs [150,151]. Collected data from the system could also serve as evidence of the efficacy of introduced preventive measures [137,152], whilst also being applicable in improving worker and employer understanding of occupational risks [156].

## Data Availability

Compensation fund annual reports: https://www.labour.gov.za/DocumentCenter/_layouts/15/touchapp.aspx?Mode=DocLibs&Page=%7B94AA76909%2DF4B3%2D4E83%2D869E%2DB2F4B16F68EF%7D (accessed on 9 December 2021). Employment statistics: http://www.statssa.gov.za/ (accessed on 9 December 2021).

## Appendix A

**Table A1:** Non-exhaustive OHS legislative history from South Africa.

Year	OHS Legislation	Commencement Date	Purpose
1941	Factories, Machinery and Building Works Act 1941	1965 *	The Act prescribed the minimum safety standards and working conditions. The Act also provided for the racial segregation of workers of different races in regards to work, recreation and eating areas for employees.
1987	Environmenmental Regulations for workplaces. Workplaces 1987	16 October 1987	Covered the requirements for thermal conditions, ventilation, noise, housekeeping, precautions against flooding and fire, lighting, and offences and penalties.
1988	General Safety Regulations 1986	30 May 1986	Prescribes the rules for activities and equipment for health and safety covering personal protective equipment and facilities; intoxication; first aid, emergency equipment and procedures; work in confined spaces; use and storage of flammable liquids.
1990	Facilities Regulations 1990	October 1990 *	Provides for sanitation, facilities for safekeeping, changing rooms, dining rooms, drinking water, prohibitions, seats, condition of rooms and facilities.
2004	Facilities Regulations 2004	3 August 2004	
1983	Machinery and Occupational Safety Act 1983	22 August 1986	Provides for the health and safety of persons at work as well as persons outside work affected by hazards arising out of the activities of persons at work; also provides for the health and safety of persons in connection with the use of plant and machinery
1994	Occupational Health and Safety Act 1993	1 January 1994	
1996	General Administrative Regulations 2003	25 June 2003	Provides administrative procedures for health and safety committees, negotiations and consultations before designation of health and safety representatives, reporting of incidents and occupational diseases, recording and investigation of incidents.
2001	Compensation of Occupational Injuries and Diseases Act 1997	6 October 1993	Provides the compensation for disablement caused by occupational injuries or diseases sustained by contracted employees in the course of their employment or for death resulting from such injuries or diseases.
2001	Regulations for Hazardous Biological Agents 2010	27 December 2001	Provides for the classification of biological agents, special measures for health and veterinary isolation facilities and occupational health programme for persons exposed to hazardous biological agents at work.
1991	Lead Regulations 1991	22 March 1991	Provides for the protection of the health and safety of any person at workplaces where lead is produced, processed, used, handled or stored in a form in which it can be inhaled, ingested or absorbed by any person in that workplace.
2002	Lead Regulations 2002	28 February 2002	
2003	Noise-Induced Hearing Loss Regulations 2003	7 March 2003	Provides for the protection of the health and safety of employers and self-employed persons carrying work exposing persons to noise at or above the 85dBA noise-rating limit
1987	Asbestos Regulations 1987	10 April 1987	Protect the health and safety of every employer and self-employed person who may expose any person to asbestos dust at the workplace
2002	Asbestos Regulations 2002	10 February 2002	
2020	Asbestos Abatement Regulations 2020	10 November 2020	
2021	Ergonomics Regulations 2018	6 December 2019	Protect the health and safety of any person who may be exposed to ergonomic risks in the workplace.
1995	Hazardous Chemical Substances Regulations 1995	25 August 1995	Regulates employers or a self-employed persons carrying out work at a workplace which may expose any person to a hazardous chemical agent at the workplace; and a manufacturer, importer, supplier or retailer of a hazardous chemical agent that is intended for use at a workplace
2021	Hazardous Chemical Agents Regulations 2021	29 March 2021	

* Date and/or month unavailable from reviewed historic documents.

## Appendix B

**Table A2:** Employment, total accidents, injuries and occupational diseases, 2001-2019 in South African industry.

	Year																			Total
	2001	2002	2003	2004	2005	2006	2007	2008	2009	2010	2011	2012	2013	2014	2015	2016	2017	2018	2019	
Overall employment	9,994,000	10,264,000	10,318,000	10,682,000	11,446,000	11,951,000	12,019,000	9,978,000	9,431,000	11,716,000	12,136,000	12,143,000	13,502,000	13,668,000	14,236,000	14,343,000	14,484,000	14,75,0000	14,695,000	-
Total occupational accidents reported	280,631	230,574	237,533	218,873	237,980	213,226	209,830	203,711	200,559	215,493	164,532	196,509	310,710	225,511	129,123	155,427	183,100	156,223	82,526	3,852,071
Total occupational safety injuries	277,270	225,885	232,515	213,515	234,158	208,662	206,110	202,268	198,664	214,382	163,177	193,930	308,131	225,511	129,123	154,949	182,309	155,623	81,995	3,808,177
Total occupational diseases	3361	4689	5018	5358	3822	4564	3720	1443	1895	1111	1475	2579	2579	0	0	478 *	791 *	600 *	531 *	44,014 *

* Difference in data from Table 1 due to carry-over cases.
